# Thyroid and Lipidic Profiles in Nicastrese Goats (*Capra hircus*) during Pregnancy and Postpartum Period

**DOI:** 10.3390/ani11082386

**Published:** 2021-08-12

**Authors:** Luigi Liotta, Arianna Bionda, Marco Quartuccio, Floro De Nardo, Rosanna Visalli, Esterina Fazio

**Affiliations:** 1 Department of Veterinary Sciences, University of Messina, Viale Palatucci, 13, 98168 Messina, Italy; luigi.liotta@unime.it (L.L.); marco.quartuccio@unime.it (M.Q.); esterina.fazio@unime.it (E.F.); 2 RARE, Italian Association of Endangered Local Breeds, Via Lorenzo Calogero, 2, 88046 Lamezia Terme, Italy; denardofloro@libero.it; 3 BIOGENE, Veterinary Diagnostic Center, Via Giacomo Leopardi, 50, 95127 Catania, Italy; biogene@biogene.it

**Keywords:** goat, pregnancy, lactation, thyroid function, lipidic panel

## Abstract

**Simple Summary:**

This study aimed to provide data about thyroid and lipid profiles in 30 Nicastrese goats along different physiological periods: before mating, during pregnancy, and in early lactation. Total triiodothyronine and thyroxine were lower in pregnant and milking goats, while BCS was lower in pregnant ones only, and total cholesterol, triglyceride, and very-low-density lipoprotein cholesterol were in milking goats only. During pregnancy and lactation, total triiodothyronine was positively correlated with free triiodothyronine and total thyroxine. These results show that under similar environmental, nutrition, and management conditions, different physiological phases play a significant role in the thyroid and lipid profiles in Nicastrese goats. These endocrine and metabolic resources could contribute to the knowledge useful for the salvage of this endangered, local, native breed.

**Abstract:**

This study aimed to determine the thyroid and lipid profiles in 30 Nicastrese goats, along different physiological periods: before mating (nonpregnant goats), during the whole pregnancy (pregnant goats), and during postpartum and early lactation (milking goats). Blood samples were collected monthly from March 2020 to January 2021. Serum thyroid-stimulating hormone (TSH), total and free triiodothyronine (T_3_, fT_3_), and thyroxine (T_4_, fT_4_) concentrations were measured using immunoenzymatic assay kits and serum lipid panels (triglyceride (TG) and total cholesterol (tCho)) by enzymatic colorimetric method; very-low-density lipoprotein cholesterol (VLDL Cho) was calculated. Pregnant and milking goats showed the lower T_3_ (*p* < 0.0002) and T_4_ (*p* < 0.0005) concentrations, with lower BCS (*p* < 0.001) only in pregnant ones. Milking goats showed tCho (*p* < 0.006) concentrations lower than nonpregnant ones, and TG and VLDL Cho (*p* < 0.001) lower than both pregnant and nonpregnant goats. T_4_:T_3_ and T_3_:fT_3_ were significantly and positively correlated in both pregnancy and lactation. Under similar environmental, nutrition, and management conditions, different physiological phases play a significant role in the thyroid and lipid profiles in Nicastrese goats. These endocrine and metabolic resources could contribute to the knowledge useful for the salvage of this endangered, local, native breed.

## 1. Introduction

Among the European countries, Italy comprises the largest number of local goat breeds (www.assonapa.com, accessed on 25 March 2021), which are mainly reared in the Alps and in the Mediterranean area, such as Southern Italy and islands. In Calabria, the farming system for goats is mainly characterized by small- to large-sized farms with either semi-sedentary farming based on natural pasture or free-ranging farming [1]. In particular, the Nicastrese goat, together with the Aspromontana and the Rustica di Calabria, represents the native Calabrian goat biodiversity. Its coat is black with a white abdomen, limbs, and part of the head, straight frontal-nasal profile, and head provided with lyre-shaped horns in both sexes. The average weight is 78 kg for adult males and 46 kg for adult females. Lactation has a duration of 180 days in primiparas with a milk production of 150 kg, while, in multiparous goats, lactation lasts from 220 to 260 days with a production of about 210 kg of milk. Milk has an average fat content of 4.30%, protein of 3.50%, and lactose of 4.70% [2]. The Nicastrese goat breed counts about 4851 individuals and 77 flocks and is bred in natural grazing land in Catanzaro, which is characterized by aromatic and functional Mediterranean plants that confer typical features to the milk and cheese. The farming system is founded on the use of natural pasture and breeding is intended for the local production of milk and cheese. The first genetic overview on the Nicastrese goat was carried out by Nicoloso et al. (2015) [3] and, recently, microbiological, physicochemical, and sensory traits of the Nicastrese goat’s raw milk and cheese have been achieved by several authors [4,5,6]. Nicastrese cheese is an artisanal cheese produced in the Calabria region, according to traditional manufacturing practices. The unique flavor of this cheese type is the result of a complex balance between volatile and non-volatile compounds, originating during the ripening process from milk’s fat, protein, and carbohydrates [6].

It is apparent that, in the main physiological phases of pregnancy and lactation, especially in the early stage, hormonal and biochemical changes dynamically occur. Thyroid hormones (THs) are known to play important roles to maintain the homeostasis of energy balance, protein metabolism, and productivity parameters, representing epigenetic signals in developmental programming [7]. Thyroid hormones assume a pivotal role in the development of the mammary gland, increasing the synthesis and release of the main milk proteins, including the α-lactalbumin [8]. Increased thyroid activity and blood hormone levels during pregnancy in all mammalian species are known [9]. In sheep, endocrine profiling revealed significantly higher values of TSH during pregnancy and T_4_ during the postpartum phase, with unchanged T_3_ concentrations in the pregnant and the postpartum phases [10].

The preservation of adequate body condition and milk supply is a fundamental aspect of early lactation; therefore, during this period, the organism directs the energy signaling toward the feed intake [11]. Moreover, lower circulating T_3_ and T_4_ concentrations were observed at the start of lactation, with an increase along with the lactation and a related decrease in the dry period [12]. It is hypothesized that a lower T_3_ concentration reduces the oxidation rate and the metabolic rate of protein and fat substrates in mammary tissue [13].

Considering lipid profiles, in the first half of pregnancy anabolic processes are predominant, while catabolic events prevail along the second half of pregnancy, with fatty acid mobilization from adipose tissue to blood of both pregnant and early lactating sheep [14,15,16]. Milk lipid metabolism involves a sophisticated regulation of fatty acid, triglyceride, and cholesterol synthesis; in goat species, the genetic and molecular mechanisms involved in this process have not been completely understood [17]. Serum total cholesterol (tChol) and triglyceride (TG), which is the principal constituent of fat tissue and milk [18], showed lower concentrations in postpartum than before, probably as a consequence of the increased energy consumption that accompanies the start of milking [19,20,21,22].

Other variables, such as season and dietary variations, have been related to the effect of the lactation stage on colostrum and milk fatty acid composition in goats [23]. A relationship between the seasonality and the bioavailability of THs for the reproductive neuroendocrine axis was also investigated in the Saanen goat, showing that the photoperiod regulates the expression of the type II deiodinase gene in the mediobasal hypothalamus [24]. Though, Cashmere goats thyroidectomized in the late breeding season advanced the onset of seasonal anestrus [25].

The transition period is the most stressful time in the productive cycle of the dairy cow and comprehensive endocrine and metabolic events were described [26]. Nevertheless, in the goat, this peculiar period received only a little attention [21], with special emphasis before mating [27], for the periparturient phase [20,28,29], and across the gestation period [19].

On the basis of this knowledge, we hypothesize, as demonstrated in other species of ruminants, that goats are able to maintain their dynamic endocrine and metabolic homeostasis along with the different physiological phases of pregnancy and lactation, according to the breed, environmental, and management stimuli. Therefore, we aimed to investigate TSH, total and free thyroid hormones, and lipid panels (triglyceride (TG), total cholesterol (tCho), and very-low-density lipoprotein cholesterol (VLDL Cho)) in nonpregnant, pregnant, and lactating Nicastrese goats. For this purpose, endogenous factors (breed, age, and physiological state), environmental factors (climate and season), and nutrition were considered.

## 2. Materials and Methods

The research complied with the guidelines of Good Clinical Practices [30]. This study was performed according to the ethical principles that have their origins in the Italian Veterinarians’ Ethical Code [31], and the Italian and European regulations on animal welfare [32]. Management and handling of the animals involved in this study were performed following the Italian and European legislation on animal welfare [33,34].

The experimental protocol was approved by the Ethical Committee of the Department of Veterinary Science of the University of Messina (code 046/2020).

### 2.1. Animals

In the present study, from March 2020 to January 2021, we monthly collected the blood of 60 multiparous Nicastrese goats, ranging in age from 3 to 4 years. All samples were taken between 7:00 and 9:00 in the morning to minimize the effect of the circadian rhythm on hormone measurements, in quiet conditions by the same operator. The sampled goats were randomly selected from a group of 400 animals, reared using semi-extensive farming practices in a commercial farm located in south Italy (Calabria region, Sila Piccola, Catanzaro), (39.048° N; 16.5653° E, 1215 m above the sea level). The breeding season of goats in Calabria occurs from September to February.

Only the data of the goats (*n* = 30) that actually gave birth were retained and analyzed so that all the results refer to the same animals in different physiological stages. All these goats had normal estrus cycles and were free of uterine disease. They became pregnant between the second half of June and the beginning of July: the first week of mating was considered the first week of gestation. Hence, at the time of the first fourth-month sampling, the pregnant goats were near the same stage of pregnancy (30 ± 5 days).

In all the included goats, pregnancy was normal, and delivery was spontaneous and eutocic. All kids were healthy and viable; they were examined by a veterinary, who diagnosed them as clinically normal. The goats breastfed their kids until weaning, which occurred at 3 months.

The goats grazed in the same pasture area, mainly consisting of natural woodland pasture, from 8 am to 4 pm, whereas they were confined in the indoor area during the night. In this area, covered with straw bedding, all the animals were fed with the same diet, in order to have similar composition and quantity of individual supplements for lactating, pregnant, and nonpregnant does, and therefore to minimize the effect of different diets on the hormonal and lipidic profiles: concentrate (on average 0.7 kg/head/day; CP: 155.1 g kg^−1^ of DM; EE: 41.2 g kg^−1^ of DM; NDF: 196.1 g kg^−1^ of DM; NEL 1.08 UFL kg^−1^ of DM) and meadow hay (on average 1 kg/head/day; CP: 110.9 g kg^−1^ of DM; EE: 25.0 g kg^−1^ of DM; NDF: 521.9 g kg^−1^ of DM; NEL 0.65 UFL kg^−1^ of DM); the water was available ad libitum.

### 2.2. Samples and Analyses

All the experimental animals were evaluated monthly for the body condition score (BCS) based on a scale of 1 (thin) to 5 (fat) in accordance with Santucci et al. (1991) [35] and submitted to individual blood sampling by vacutainer tubes from the jugular vein.

Blood samples, of approximately 5 mL, were placed in a sterile glass tube to obtain serum samples for biochemical and hormonal analyses. For this purpose, serum was centrifugated within 60 min of collection and refrigerated at 4 °C for chemical and hormonal analyses, performed within one week after collection, at the Veterinary Diagnostic Center BIOGENE (Catania, Italy).

Serum TSH, T_3_, fT_3_, T_4_, and fT_4_ concentrations were assessed using a homologous solid-phase, two-site chemiluminescent immunometric assay (Immulite^®^ 2000, Siemens Medical Solutions-Diagnostics-USA), according to the manufacturer’s instructions.

TSH intra-assay and inter-assay coefficient of variations (CVs) were 5.5% and 9.5% at TSH concentrations of 0.2 and 2.35 ng/mL, respectively. The sensitivity of the assay was 0.01 ng/mL.

T_3_ intra-assay and inter-assay CVs were 12% and 5.5% at T_3_ concentrations of 73 ng/dL and 171 ng/dL, respectively. The sensitivity of the assay was 19 ng/dL.

fT_3_ intra-assay and inter-assay CVs were 9.1% and 5.4% at fT_3_ concentrations of 3.2 pg/dL and 13 pg/dL, respectively. The fT_3_ sensitivity of the assay was 1.0 pg/mL.

T_4_ intra-assay and inter-assay CVs were 11.1% and 5.6% at T_4_ concentrations of 1.8 µg/dL and 16 µg/dL, respectively. The sensitivity of the assay was 0.3 µg/dL.

fT_4_ intra-assay and inter-assay CVs were 3.0% and 10.2% at fT_4_ concentrations of 4.82 ng/dL and 0.51 ng/dL, respectively. The sensitivity of the assay was 0.11 ng/dL.

Lipid panels (triglyceride [TG] and total cholesterol [tCho]) were analyzed by enzymatic colorimetric method, using BT3500 Biotechnic Instruments; very-low-density lipoprotein cholesterol (VLDL Cho) content was calculated as triglycerides divided by 5.

### 2.3. Statistical Analysis

All the statistical analyses were performed using JMP^®^ 16 (SAS Institute Inc., Cary, NC, USA). Descriptive statistics were generated for all the examined parameters. Results were reported as mean ± standard deviation of the mean (sd). A mixed model was used to compare all the examined parameters among the different physiological phases (pregnancy, lactation, and nonpregnancy) and the different months within each phase; animals were considered as a random effect in order to account for the correlation between the repeated measurements within each subject [36]. To determine which groups differed from each other, the Tukey’s HSD post-hoc test was applied. Pearson’s correlation coefficients (r) were used to measure the relationships between all measured parameters. The significance threshold was set to the conventional value of 0.05.

## 3. Results

In the present study, the BCS, circulating thyroid-stimulating hormone (TSH), total and free triiodothyronine (T_3_, fT_3_) and thyroxine (T_4_, fT_4_) concentrations, and lipid panels (triglyceride [TG], total cholesterol [tCho], and very-low-density lipoprotein cholesterol [VLDL Cho]) measured during different physiological phases (nonpregnancy, pregnancy, and lactation) were described as mean ± sd and ranges were reported in Table 1, Table 2, Table 3 and Table 4.

Compared to the nonpregnant period, pregnant and lactating goats showed the lower T_3_ (*p* < 0.0002) and T_4_ (*p* < 0.0005) concentrations, with lower BCS (*p* < 0.001) only in pregnant goats (Table 1). The lactation period showed lower tCho (*p* < 0.006) concentrations than the nonpregnant period, and lower TG and VLDL Cho (*p* < 0.001) than both the pregnant and nonpregnant periods (Table 1).

## 4. Discussion

### 4.1. Thyroid Profile

The results obtained from the goats included in the present study for TSH, T_3_, fT_3_, T_4_, and fT_4_ concentrations were in accordance with published physiological ranges reported in adult nonpregnant goats for T_3_ (0.59–1.35 ng/mL) and T_4_ (6.10–8.15 µg/dL) [37]; in pregnant and postpartum goats for T_3_ (17.11 ± 5.01 nmol/L), T_4_ (11.25 ± 1.33 ng/mL) [38], fT_3_ (1.02–1.29 pg/mL), and fT_4_ (0.51–1.71 ng/dL and 31.5–32.7 pmol/L) [39,40]; and in nonpregnant, pregnant, and postpartum sheep for TSH values (0.50 ± 0.05 µg/mL, 0.70 ± 0.014 µg/mL, and 0.24 ± 0.10 µg/mL) [10]; nevertheless, some variation might be due to differences in the techniques used [41]. Data obtained excluded the possible influence of circadian thyroid rhythms because the blood sampling was always performed at the same time for all the goats in nonpregnant, pregnant, and postpartum phases. In addition, the significant differences of T_3_ and T_4_ concentrations among nonpregnant, pregnant, and lactating phases, with the lowest values of T_3_ and T_4_ in the early lactating goats and their highest values in nonpregnant goats, confirm the trend of T_3_ observed by Karapehlivan et al., (2007) and Antunović et al., (2011) [42,43] in the ewes during different physiological status, and of T_3_ and T_4_ reported by Pezzi et al., (2003) [12] in lactating cows. These last authors showed lower circulating THs concentrations at the start of lactation, with an increase during lactation and, subsequently, a decrease during the dry period. On the other hand, the trend of T_3_ and T_4_ described in Nicastrese goats do not confirm previous data observed in nonpregnant, pregnant, and postpartum phases in sheep [10] and goats [9,37,39], with increased thyroid activity and circulating THs concentrations during pregnancy and postpartum phases.

Specifically, the lower T_3_ and T_4_ concentrations in pregnant and lactating phases than in the nonpregnant period could be indicative of their higher synthesis, release, and related use during these dynamic periods, according to the same trend observed for lipid panels. It is possible to presume that the thyroid response to pregnancy and early lactation in the regulation of energy homeostasis incorporates hormonal signals that initiate energy preservation. These results confirmed that pregnancy and the early lactation stage represent a very demanding physiological state of the specimen when nutritional requirements are increased [44]. Hence, in the pregnant and lactating periods, the lowest T_3_ and T_4_ concentrations are probably due to their increased demand and use for anabolic processes and the requirements of the higher metabolic and oxidation rates, as reported in ruminant species [9], according to the productivity parameters [45]. This would confirm that, in mammals, pregnancy influences the thyroid hormone utilization rates [46], possibly as a consequence of the high thyroidal activity of the fetus simultaneously with negative feedback on the maternal total iodothyronine concentrations. Given the positive and significant correlation between T_3_ and T_4_ observed in pregnant goats, it is reasonable to suppose that peripheral tissues’ conversion of T_4_ to T_3_ is enhanced by the maternal, and probably also the fetal, thyroid synthesis. These results confirm that the active metabolic role of these iodothyronines, already reported in growing foals [47], mares [48,49], and donkeys [50], also concerns goats.

The significantly lowest T_3_ and T_4_ values observed during the early lactation/postpartum phase confirmed the consistent role played by THs in the development of the mammary gland and related synthesis and release of milk proteins [8]; in fact, these thyroid effects were corroborated by a positive and significant correlation between T_3_ and T_4_ changes recorded in this dynamic phase. Moreover, the lower THs concentrations might be due to the preparation of the mammary activity and the start of the lactogenesis phase, which require a large use of them.

In addition, the significant correlation between T_3_ and fT_3_ values observed in both pregnancy and lactating phases confirms previous data observed in growing foals, reporting that changes of total iodothyronine concentrations generally follow those of the free quota [47].

Although no significant differences among June-November (35–135 days of pregnancy) were recorded for TSH and T_3_, data obtained reported the highest values of these hormones in August, as well as observed for the significant highest values of T_4_ and fT_4_, with the lowest T_3_, and significant lowest fT_3_ and T_4_ values in September. The environmental temperature represents the major exogenous regulator of thyroid activity [51], so an inverse relationship between ambient temperature and circulating thyroid hormones was described in both goats [52,53] and sheep [54,55]. In fact, the seasonal thyroid pattern often showed maximal values during winter and minimal during summer [56,57]. Nevertheless, contrasting data were observed [24,58]; moreover, in the Sahel desert, plasma T_3_ and T_4_ concentrations showed significant rise at the onset of the humid warm season (June) [59], and in the Umbrian population, goats showed high thyroid hormones concentrations in spring, according to the increasing day length, and low in autumn, with a decreasing day length [37]. The results of the present study partially confirm data previously observed by different authors, with the highest THs in January for lactating goats, and lowest in September for pregnant goats. Some differences could be explained by the influence of physiological or geographic factors and different management. Moreover, the data obtained exclude the possible influence of thyroid rhythms because blood sampling was always performed at the same time. The seasonal trend of THs could indicate that when the temperature ranges are not extreme, as in our experimental conditions, in which environmental temperature ranged between 2 °C and 18 °C, the effect of the physiological phase on the thyroid profile is observed. Further studies may highlight the extent of the influence of seasonality on hormonal profiles during different physiological stages. 

We concluded that total thyroid hormones during pregnancy and lactation phases are correlated in Nicastrese goats and that both these phases can promote a consistently lower T_3_ and T_4_ concentration, recognizing the maternal increased metabolic activities as a concomitant cause of their lower concentrations.

Pregnancy and early lactation had a major influence on the intensity of metabolism and on circulating lipidic parameters, as speculated by the existence of negative significant correlations among fT_4_ and tCho, VLDL Cho, and TG (during the pregnancy period) and negative between fT_3_ and Cho, but positive between T_4_ and tCho (during the lactating period). These correlations could support the knowledge that thyroid hormones affect cholesterol metabolism [60,61], partially confirming data previously reported by Gueorguieva and Gueorguieva (1997) [62], who obtained a consistently negative correlation among T_3_, T_4_, and serum cholesterol in dairy cows; hence, the seasonal change observed in serum tCho, TG, and VLDL Cho may be partly due to changes in thyroid hormones.

### 4.2. Body Condition Score

The significantly lower BCS during pregnancy than nonpregnant goats, with a biphasic trend represented by the highest score from 35 to 95 days and the lowest score at the end of pregnancy, at 115 and 135 days, was speculated to be the result of the start of milking and the increased body energy consumption, according to Watson et al. (1993) [63]. In this phase, lipid turnover is enhanced due to the changes in maternal metabolism and the functional differentiation of tissues, particularly in the reproductive and mammary systems; hence, the existence of significant differences of tCho, TG, and VLDL Cho concentrations, with the lowest values in the early lactating phase and the highest values in both nonpregnant and pregnant phases, showed a significant influence of the physiological period on the lipid homeostasis. In addition, the pregnant period seems to have a major influence on the intensity of metabolism and on BCS, as speculated by the existence of significant negative correlations among BCS and Cho, TH, and VLDL Cho.

### 4.3. Lipidic Panels

The overall mean tCho, TG, and VLDL Cho ranges calculated in this study are in accordance with physiological ranges reported for clinically healthy nonpregnant goats (tCho: 53.9 ± 1.59 mg/dL), pregnant goats (tCho: 82.08 ± 1.58 mg/dL, TG: 85–100 mg/dL, and VLDL Cho: 17–20 mg/dL), and adult female goats (2–4 years) (tCho: 113.64 ± 23.03 mg/dL) [19,20,21,64,65], and confirm the significant increase of serum tCho, TG, and VLDL Cho concentrations in small ruminants, from the last gestational phase, peaking at 115 and 135 days, compared to the early and mild gestational period [19]. Likewise, the increased serum tCho, TG, and VLDL Cho concentrations in pregnant goats are in accordance with data observed in small ruminants, in which this increase was attributed to an increased hepatic TG synthesis [10,19,21,66]. Moreover, the lower Cho, TG, and VLDL Cho concentrations described during the first 35–95 days of pregnancy than in the second half of pregnancy and in lactating reference goats are in line with data previously recorded in the Sahel goat by Waziri et al., (2010) [19]. In addition, the lower serum tCho, TG, and VLDL concentrations in the postpartum/early lactation phase were similar to those reported in early and mild gestational phases and were attributed to the moderately increased lipoprotein lipase activity, occurring with the induction of the enzyme in mammary tissue to provide for milk-fat synthesis. Although the fat content in goat’s colostrum and milk during the first month of lactation is higher than in mature milk [23], lactation would require an increased supply of fatty acids for lipidic synthesis, confirming the significant lowest lipidic panels observed during the early stage of lactation.

Decreases in T_3,_ T_4_, tCho, TG, and VLDL Cho concentrations have been related to milk production, probably reflecting the difference in nutrient availability and metabolism of lactating and nonlactating (nonpregnant) goats. Thus, under similar environmental and body conditions, nutrition regime, and management system, the lactation stage appears to play a significant role on the thyroid and lipidic profiles in Nicastrese goats.

The physiological values obtained in this study may be used as additional resources to evaluate thyroid and lipidic patterns in nonpregnant, pregnant, and lactating Nicastrese goats, and to generate desirable observations, especially when the different physiological conditions are considered. Likewise, with the constant pressure of the small ruminant industry to develop and optimize methods to promote the milk derivates productions, it appears that continuing the research on this topic remains fully justified.

## 5. Conclusions

In this study, we consolidate the small amount of previously reported physiological data with an additional result that reflects crosstalk between hormonal, metabolic patterns, and functional stage. These data contribute to the overall knowledge on the physiological thyroid and lipidic patterns of Nicastrese goats, mainly intended for milk production for local cheese manufacturing.

These results could be considered as an original set of knowledge useful for the salvage of this endangered, local, native breed, encouraging Calabrian farmers and others to grow and complete the development of the young male and female stocks of goats on their farms of origin. This new productive strategy could increase farmers’ incomes, especially if this milk and cheese are promoted as ‘local niche’ food obtained from autochthonous endangered local goats, whose husbandry is actively contributing to the maintenance of the fragile Calabrian environment. Some goat populations were saved from the risk of extinction thanks to the initiative of few stockmen who transferred some of their flocks to the Italian region, where the goats reproduced without the risk of contamination by other breeds. Under a conservation program, the preservation of rare genotypic specimens could be feasible.

Our findings should be considered as an encouragement to further research addressing this specific issue in more detail.

## Figures and Tables

**Table 1:** Circulating thyroid-stimulating hormone (TSH), total and free triiodothyronine (T_3_, fT_3_), total and free thyroxine (T_4_, fT_4_) concentrations, body condition score (BCS), and lipidic panels (mean ± sd) and min and max values in different physiological periods of 30 Nicastrese goats. Within each row, different superscript letters indicate significantly different values.

Periods	Nonpregnancy	Pregnancy	Lactation	*p*-Value
TSH (ng/mL)	0.17 ± 0.07	0.16 ± 0.06	0.14 ± 0.04	0.2290
	0.10–0.38	0.10–0.34	0.10–0.25	
T_3_ (ng/dL)	108.27 ± 30.60 ^A^	90.05 ± 24.13 ^B^	75.22 ± 13.59 ^B^	0.0002
	59.5–211.00	54.2–156.00	50.20–102.00	
fT_3_ (pg/mL)	1.89 ± 0.52	1.80 ± 0.53	1.69 ± 0.37	0.3943
	1.02–2.79	1.03–2.98	1.03–2.64	
T_4_ (µg/dL)	7.18 ± 1.58 ^A^	5.76 ± 1.44 ^B^	5.39 ± 1.07 ^B^	0.0005
	3.65–10.10	3.06–8.80	3.04–8.15	
fT_4_ (ng/dL)	1.01 ± 0.27	0.91 ± 0.29	1.06 ± 0.22	0.1462
	0.51–1.65	0.45–1.68	0.62–1.52	
BCS (score 1–5)	2.95 ± 0.1 ^A^	2.71 ± 0.29 ^B^	2.84 ± 0.20 ^A,B^	0.0012
	2.75–3.00	2.00–3.00	2.50–3.00	
Cholesterol (mg/dL)	99.57 ± 24.40 ^A^	91.66 ± 24.73 ^A,B^	79.33 ± 12.08 ^B^	0.0060
	50.00–157.00	57.00–159.00	54.00–103.00	
Triglyceride (mg/dL)	59.2 ± 33.87 ^A^	53.31 ± 32.48 ^A^	26.44 ± 3.95 ^B^	<0.0001
	20.00–169.00	20.00–145.00	20.00–39.00	
VLDL Cho (mg/dL)	11.84 ± 6.77 ^A^ 4.00–34.00	10.66 ± 6.50 ^A^ 4.00–30.00	5.29 ± 0.79 ^B^ 4.00–8.00	<0.0001

The comparison among the different months of the nonpregnant period, ranging from March to May (Table 2), showed the higher T_3_ (*p* < 0.01), fT_3_ (*p* < 0.0003), and fT_4_ (*p* < 0.0008) concentrations in both March and April than May; higher T_4_ (*p* < 0.0001), tCho (*p* < 0.0007), TG (*p* < 0.0001) and VLDL Cho (*p* < 0.0001) concentrations in both April and May than March.

**Table 2:** Circulating thyroid-stimulating hormone (TSH), total and free triiodothyronine (T_3_, fT_3_), total and free thyroxine (T_4_, fT_4_) concentrations, body condition score (BCS), and lipidic panels (mean ± sd) in nonpregnant periods of 30 Nicastrese goats. Within each row, different superscript letters indicate significantly different values. Temperature measurements refer to the environmental temperature at the sampling time.

Months Temperature	March (5 °C)	April (7 °C)	May (9 °C)	*p*-Value
TSH (ng/mL)	0.17 ± 0.08	0.19 ± 0.07	0.15 ± 0.06	0.1684
T_3_ (ng/dL)	115.96 ± 39.01 ^A^	116.42 ± 27.42 ^A^	93.13 ± 17.54 ^B^	0.0132
fT_3_ (pg/mL)	2.09 ± 0.49 ^A^	2.1 ± 0.38 ^A^	1.46 ± 0.43 ^B^	0.0003
T_4_ (µg/dL)	6.21 ± 1.52 ^B^	7.49 ± 1.45 ^A^	7.85 ± 1.35 ^A^	<0.0001
fT_4_ (ng/dL)	1.12 ± 0.29 ^A^	1.05 ± 0.23 ^A^	0.86 ± 0.22 ^B^	0.0008
BCS (score 1–5)	2.92 ± 0.12	2.94 ± 0.11	3.00 ± 0.00	0.1573
Cholesterol (mg/dL)	83.67 ± 14.95 ^B^	107.82 ± 28.48 ^A^	107.92 ± 21.45 ^A^	0.0007
Triglyceride (mg/dL)	25.67 ± 6.05 ^B^	75.09 ± 18.72 ^A^	78.17 ± 36.31 ^A^	<0.0001
VLDL Cho (mg/dL)	5.13 ± 1.21 ^B^	15.02 ± 3.74 ^A^	15.63 ± 7.26 ^A^	<0.0001

The comparison among the different months of pregnancy, ranging from June (35 days) to November (135 days) (Table 3), showed the significant highest values of T_4_ (*p* < 0.0001) and fT_4_ (*p* < 0.0002) in August, of fT_3_ (*p* < 0.001) in October, and the lowest fT_3_ (*p* < 0.001) and T_4_ (*p* < 0.0001) values in September. Although no significant differences between June and November for TSH and T_3_ were recorded, data obtained reported their highest values in August, and the lowest values in July for TSH and in September for T_3_. BCS showed a higher score from June to September (*p* < 0.0001) than October and November (Table 3). tCho, TG, and VLDL Cho concentrations showed a biphasic trend, with the lower values from June to September (*p* < 0.0001) than October and November (Table 3).

**Table 3:** Circulating thyroid-stimulating hormone (TSH), total and free triiodothyronine (T_3_, fT_3_), total and free thyroxine (T_4_, fT_4_) concentrations, body condition score (BCS), and lipidic panels (mean ± sd) in pregnant periods of 30 Nicastrese goats. Within each row, different superscript letters indicate significantly different values. Temperature measurements refer to the environmental temperature at the sampling time.

Months Temperature	June (13 °C)	July (16 °C)	August (18 °C)	September (15 °C)	October (12 °C)	November (9 °C)	*p*-Value
Days	35	55	75	95	115	135	
TSH (ng/mL)	0.16 ± 0.05	0.13 ± 0.04	0.19 ± 0.07	0.16 ± 0.06	0.16 ± 0.06	0.17 ± 0.05	0.2364
T_3_ (ng/dL)	100.94 ± 35.33	82.59 ± 25.95	108.26 ± 35.15	78.93 ± 16.74	98.3 ± 22.64	83.96 ± 11.55	0.0062
fT_3_ (pg/mL)	2.01 ± 0.57 ^A,B^	1.87 ± 0.64 ^A,B^	2.04 ± 0.58 ^A,B^	1.51 ± 0.30 ^B^	2.10 ± 0.55 ^A^	1.53 ± 0.39 ^A,B^	0.0013
T_4_ (µg/dL)	6.35 ± 1.69 ^A,B^	6.28 ± 1.68 ^A,B^	6.72 ± 1.24 ^A^	4.63 ± 0.82 ^B^	6.28 ± 1.20 ^A^	5.47 ± 1.39 ^A,B^	<0.0001
fT_4_ (ng/dL)	1.07 ± 0.32 ^A,B^	1.21 ± 0.25 ^A^	1.23 ± 0.21 ^A^	0.81 ± 0.22 ^B,C^	0.84 ± 0.27 ^B,C^	0.74 ± 0.19 ^C^	0.0002
BCS (score 1–5)	3.00 ± 0.50 ^A^	3.00 ± 0.50 ^A^	3.00 ± 0.50 ^A^	2.76 ± 0.19 ^A^	2.57 ± 0.21 ^B^	2.45 ± 0.29 ^B^	<0.0001
Cholesterol (mg/dL)	71.71 ± 9.45 ^B^	78.13 ± 9.86 ^B^	70.14 ± 9.60 ^B^	74.71 ± 11.96 ^B^	118.33 ± 21.71 ^A^	103.82 ± 17.47 ^A^	<0.0001
Triglyceride (mg/dL)	26.43 ± 6.92 ^C^	33.50 ± 12.24 ^C^	29.43 ± 3.60 ^C^	25.94 ± 3.47 ^C^	95.22 ± 24.98 ^A^	66.53 ± 18.12 ^B^	<0.0001
VLDL Cho (mg/dL)	5.29 ± 1.38 ^C^	6.69 ± 2.45 ^C^	5.89 ± 0.72 ^C^	5.19 ± 0.69 ^C^	19.04 ± 5.01 ^A^	13.31 ± 3.62 ^B^	<0.0001

Significant and positive correlations were observed between T_4_:T_3_ (r = 0.54; *p* < 0.0001), T_3_:fT_3_ (r = 0.67; *p* < 0.0001), and fT_4_:BCS (r = 0.48; *p* < 0.0003); in addition, fT_4_ showed negative correlations with tCho (r = −0.29; *p* < 0.03), TG (r = −0.27; *p* < 0.05), and VLDL Cho (r = −0.27; *p* < 0.05). BCS was also negatively correlated with tCho (r = −0.56; *p* < 0.0001), TG (r = −0.48; *p* < 0.0001), and VLDL Cho (r = −0.48; *p* < 0.0001). Significant changes were observed in the postpartum phase, along the first 40, 60, and 80 days of lactation, only for T_3_ concentrations, with the highest values in January and the lowest in December (*p* < 0.04) (Table 4). Significant and positive correlations were observed between T_4_:T_3_ (r = 0.45; *p* < 0.006) and T_3_:fT_3_ (r = 0.48; *p* < 0.003), while negative between fT_3_:Cho (r = −0.40; *p* < 0.01).

**Table 4:** Circulating thyroid-stimulating hormone (TSH), total and free triiodothyronine (T_3_, fT_3_), total and free thyroxine (T_4_, fT_4_) concentrations, body condition score (BCS), and lipidic panel (mean ± sd) in lactating periods of 30 Nicastrese goats. Within each row, different superscript letters indicate significantly different values. Temperature measurements refer to the environmental temperature at the sampling time.

Months Temperature	November (10 °C)	December (5 °C)	January (2 °C)	*p*-Value
Days	40	60	80	
TSH (ng/mL)	0.13 ± 0.04	0.15 ± 0.03	0.13 ± 0.04	0.2770
T_3_ (ng/dL)	74.26 ± 13.6 ^A,B^	71.38 ± 12.99 ^B^	80.02 ± 13.76 ^A^	0.0413
fT_3_ (pg/mL)	1.66 ± 0.31	1.67 ± 0.42	1.74 ± 0.40	0.6838
T_4_ (µg/dL)	5.06 ± 0.77	5.43 ± 1.16	5.67 ± 1.23	0.0779
fT_4_ (ng/dL)	1.01 ± 0.19	1.11 ± 0.17	1.04 ± 0.29	0.2306
BCS (score 1–5)	2.83 ± 0.21	2.88 ± 0.19	2.81 ± 0.21	0.6001
Cholesterol (mg/dL)	77.46 ± 11.68	82.15 ± 11.78	78.38 ± 13.18	0.3271
Triglyceride (mg/dL)	25.54 ± 4.03	26.69 ± 5.02	27.08 ± 2.53	0.6156
VLDL Cho (mg/dL)	5.11 ± 0.81	5.34 ± 1.01	5.42 ± 0.51	0.6156

## Data Availability

The datasets generated during and/or analyzed during the current study are available from the corresponding author on reasonable request.
